# Glucose Control Has an Impact on Cerebral Blood Flow Alterations in Chronic Tinnitus Patients

**DOI:** 10.3389/fnins.2020.623520

**Published:** 2021-02-04

**Authors:** Wenqing Xia, Jinluan Cui, Yong Luo, Jin-Jing Xu, Huiyou Chen, Xindao Yin, Jianhua Ma, Yuanqing Wu

**Affiliations:** ^1^Department of Endocrinology, Nanjing First Hospital, Nanjing Medical University, Nanjing, China; ^2^Department of Radiology, Nanjing First Hospital, Nanjing Medical University, Nanjing, China; ^3^Department of Otolaryngology, Nanjing First Hospital, Nanjing Medical University, Nanjing, China

**Keywords:** tinnitus, type 2 diabetes mellitus, cognitive impairment, cerebral blood flow, magnetic resonance imaging

## Abstract

**Purpose:**

Both tinnitus and type 2 diabetes mellitus (T2DM) are linked with cognitive decline and brain dysfunction. This study used arterial spin labeling (ASL) perfusion functional magnetic resonance imaging (fMRI) to examine the abnormal cerebral blood flow (CBF) patterns existed in tinnitus patients and potential relationships between the abnormal CBF and cognitive performance. The impact of T2DM on CBF alterations in tinnitus patients was further explored.

**Methods:**

Sixty tinnitus patients and 40 non-tinnitus subjects were recruited. CBF images were collected and analyzed using ASL perfusion fMRI. Brain regions with CBF alterations between tinnitus patients and non-tinnitus controls were identified by one-way analysis of variance. Interaction effects between tinnitus and T2DM for CBF changes were also selected. Then, correlation analyses were calculated to specify the link between CBF changes and cognitive performance and between CBF changes and diabetic characteristics.

**Results:**

Tinnitus patients showed decreased CBF, primarily in the auditory area and default mode network (DMN), compared with non-tinnitus controls. Decreased CBF in these regions was correlated with executive function and attention. The interaction effect between tinnitus and T2DM was significant in the right medial prefrontal gyrus. Additionally, CBF in the right medial prefrontal gyrus was correlated with tinnitus distress and cognitive performance. In tinnitus patients, Hemoglobin A_1c_ was associated with CBF in the right medial prefrontal gyrus.

**Conclusion:**

Tinnitus affects brain perfusion in the auditory area and DMN. T2DM and uncontrolled glucose levels may aggravate a CBF decrease in tinnitus patients. These new findings implied that tinnitus patients may benefit from blood glucose control in terms of their cognitive function and tinnitus distress.

## Introduction

Tinnitus is a common audiological disorder that affects approximately 10–15% of adult populations (Langguth et al., 2013; Nemholt et al., 2015). Metabolic disorders, such as hyperinsulinemia and diabetes, are considered as risk factors of tinnitus symptoms or comorbidities and have significant relationships with the tinnitus distress, with or without hearing loss (Taneja, 2017). Tinnitus as a symptom is mostly observed in disorders involving metabolic disturbances and diabetes mellitus (DM) (Nemati et al., 2018). Conversely, higher blood glucose level will increase the burden of tinnitus distress.

Numerous studies have demonstrated poorer cognitive performance, especially on control of attention, executive, and memory in tinnitus patients compared to individuals without tinnitus (Das et al., 2012; Pierce et al., 2012; Heeren et al., 2014); however, there are still a few investigations that reported no influence of tinnitus in the cognitive function (Husain et al., 2015). Interestingly, both clinical and animal studies suggest that type 2 DM (T2DM) is a modifiable and independent causal risk factor for the development and progression of cognitive impairment (Zuloaga et al., 2016; Pal et al., 2018). Therefore, T2DM is a potential risk factor for the cognitive decline observed in tinnitus patients. The precise correlation between hyperglycemic state and brain functioning has not been illustrated in tinnitus patients.

Cognitive deterioration may be affected by a declined CBF as a result of brain ischemia and energy depletion (Ogoh, 2017). Cerebral blood flow (CBF) can be evaluated using single-photon emission computed tomography (SPECT). Current researches using SPECT shows that tinnitus patients exhibit decreased CBF in certain brain areas (Farhadi et al., 2010; Laureano et al., 2014). Arterial spin labeling (ASL) is a non-invasive functional magnetic resonance imaging (fMRI) technique for measuring regional CBF that uses magnetically labeled blood water as an endogenous tracer (Detre et al., 1992). With higher resolution and accurate localization, ASL perfusion fMRI has been applied to detect early stages of dementia patients (Binnewijzend et al., 2013; Dolui et al., 2020), but has never been used to evaluate CBF in tinnitus patients. Regarding T2DM, our previous study shows that T2DM patients exhibited reduced CBF in widespread brain regions, primarily in the visual area and the default mode network (DMN) (Xia et al., 2015), which is later confirmed by further studies (Bangen et al., 2018; Hu et al., 2019). Further research is needed to investigate whether brain areas show CBF alterations in the tinnitus patients and to determine whether T2DM is involved in the CBF fluctuation in these regions.

Hence, hypothesis was raised that patients with tinnitus would exhibit abnormal CBF and cognitive decline compared with non-tinnitus subjects and that the comorbidity of T2DM would aggravate the abnormal brain perfusion. We also intended to explore whether diabetic characteristics would be correlated with higher CBF values in tinnitus patients.

## Materials and Methods

### Participants

Study protocol was approved by the Research Ethics Committee of the Nanjing Medical University prior to study initiation. All the subjects provided written informed consent before any study procedures.

Through community health screening and newspaper advertisements, 60 patients with tinnitus and 40 non-tinnitus controls, who were all right-handed and completed at least 6 years of education, were recruited (matched for age, sex, and education) from March 2016 to November 2019. All tinnitus patients reported their tinnitus as bilateral or originating within the head. According to the World Health Organization 1999 criteria for T2DM diagnosis, 25 tinnitus patients were diagnosed with T2DM. Participants who had hearing loss (defined as thresholds ≥25 dB HL) at the frequencies of 0.25, 0.5, 1, 2, 4, and 8 kHz were excluded. Participants with hyperacusis were excluded according to Hyperacusis Questionnaire (Khalfa et al., 2002). In addition, according to previous diagnostic criteria (Lopez-Escamez et al., 2015), patients with Ménière diseases were excluded. Subjects were also excluded if they had objective tinnitus, severe visual loss, a history of stroke, major medical illness, neurological or psychiatric illness such as Alzheimer disease, head injury, anemia, Parkinson disease, epilepsy, and major depression, magnetic resonance imaging (MRI) contraindications, severe heart diseases, and damaged liver/kidney function.

### Clinical Data

Demographic information was collected prior to the MRI scan. Hearing thresholds were assessed by pure tone audiometry by an experienced otolaryngologist. The tinnitus distress was assessed by the Iowa version of the Tinnitus Handicap Questionnaire (THQ) (Kuk et al., 1990). After an overnight fast, venous blood samples were collected. Fasting blood glucose, hemoglobin A_1c_ (HbA_1c_), and blood lipid levels were assessed. Cognitive performance was evaluated by the cognitive tests in a fixed order, including the Mini Mental State Examination (MMSE), Rey–Osterrieth Complex Figure Test (CFT), Auditory Verbal Learning Test, Clock Drawing Test, and Trail Making Test (TMT) Parts A and B.

### Magnetic Resonance Imaging Data Acquisition

All participants were scanned using a 3.0-T MRI scanner (Ingenia, Philips Medical Systems, Netherlands). The participants were instructed to rest quietly with eyes closed and avoiding either falling asleep or making a sudden head motion and to not think of anything in particular. Three-dimensional T1-weighted images were acquired using the parameters as follows: repetition time (TR) = 8.1 ms, echo time (TE) = 3.7 ms, thickness = 1 mm, slices = 170, gap = 0 mm, flip angle (FA) = 8°, field of view (FOV) = 256 mm × 256 mm, and acquisition matrix = 256 × 256. ASL images were obtained with a pseudocontinuous ASL sequence with a two-dimensional fast spin-echo acquisition and background suppression using the parameters as follows: TR = 4,000 ms, TE = 11 ms, slice thickness = 4 mm, label duration = 1,650 ms; postlabel delay = 2,000 ms, FA = 90°, FOV = 220 mm × 220 mm, slices thickness = 4 mm, gap = 0.4 mm, and matrix = 64 × 64.

### Imaging Data Processing

To estimate the whole-brain volumes, a voxel-based morphometry (VBM) approach was performed using the VBM8 toolbox^1^. We used DARTEL to improve intersubject registration of the structural images. Briefly, cerebral tissues were segmented into gray matter (GM), white matter (WM), and cerebrospinal fluid by a unified segmentation algorithm (Ashburner and Friston, 2005). Resulting GM and WM images were then normalized to the Montreal Neurological Institute (MNI) template. Smoothing was followed by using a 6-mm full width at half maximum (FWHM) Gaussian kernel. The final voxel-wise GM volume maps were later entered as covariates in the ASL analysis.

Using the ASL Perfusion MRI Signal Processing Toolbox (ASLtbx), which is based on SPM12^2^ (Wang, 2012), the ASL data were preprocessed to generate CBF maps. Images were first rearranged and adjusted to correct head movement. Then, a non-linear transformation was performed on the CBF images of non-tinnitus subjects, which were co-registered with the positron emission tomography (PET) perfusion template in MNI space. The MNI-standard CBF template was defined as the average co-registered CBF images of the control group. Next, the CBF images of all subjects were co-registered to the MNI-standard CBF template. Every co-registered CBF was removed from the non-brain tissue, followed by a spatial smoothing using a 6-mm FWHM isotropic Gaussian. Normalization was finally performed. The CBF was divided per voxel by the average CBF (Aslan and Lu, 2010). Two tinnitus patients and one non-tinnitus control were excluded from the study because of head movement exceeding 2.0 mm of maximum translation in any of the *x*, *y*, and *z* directions or 2.0° of the maximum rotation around the three axes.

### Statistical Analysis

Clinical measures were analyzed using Statistical Package for the Social Sciences (SPSS) statistics software package version 20.0. Differences between the patients with tinnitus and non-tinnitus subjects were calculated. For normally distributed variable, a Student *t* test was used. For asymmetrically distributed variable, a non-parametric Mann–Whitney *U* test was used. For categorical variable, a *χ*^2^ test was used. The statistical significance level was set at *p* < 0.05, two-tailed.

A one-way analysis of variance (ANOVA) was then performed to determine between-group differences in brain volumes, with age, gender, and education level as the nuisance covariates. Between-group differences in CBF were also calculated via one-way ANOVA in SPM12, with age, gender, and education level as the nuisance covariates. Significant thresholds were corrected using false discovery rate criterion and set at *p* < 0.01. A full-factorial model was utilized to detect potential interaction effects between tinnitus and diabetes on CBF differences. Significant thresholds were corrected using cluster-level family-wise error (FWE), and the threshold was set at *p* < 0.01.

The relationships between abnormal CBF in certain brain areas and THQ scores, cognitive performance, and glucose levels were further calculated. First, the brain regions that showed significant differences between the two groups were extracted, and the mean *z* values of abnormal CBF region mask were calculated within each subject. Then, Pearson correlation analysis between the mean *z* values and THQ scores, cognitive performance, and glucose characteristic was performed using SPSS software. Partial correlations were calculated after correction for age, gender, and education. *P* < 0.05 was considered statistically significant.

## Results

**FIGURE 1:**
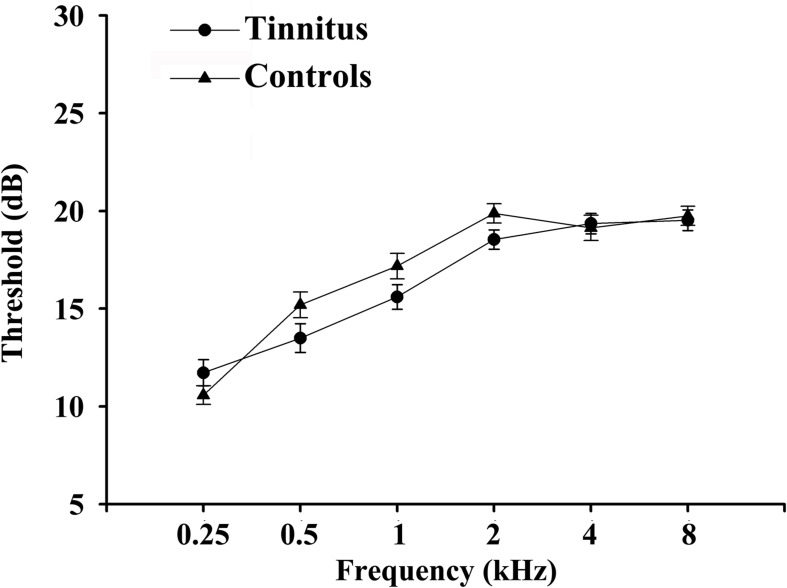
Average hearing thresholds of chronic tinnitus patients and non-tinnitus controls. Data are presented as mean ± SEM.

A set of Chinese Han population was tested in the current study. The average age for tinnitus was 49.81 ± 10.57 years, and duration of tinnitus was 37.07 ± 34.40 months. There are no significant differences between tinnitus patients and controls in terms of age, gender, and education level. Key diabetes characteristics (i.e., HbA_1c_, fasting glucose, and lipid levels) also did not differ meaningfully between the two groups. All subjects exhibited normal performance according to MMSE score. We detected a trend for a difference in TMT-A and TMT-B scores (*p* < 0.001) between groups (Table 1). There were no significant differences of auditory thresholds between tinnitus patients and non-tinnitus controls (Figure 1).

**TABLE 1:** Demographics and cognitive characteristics of tinnitus patients and non-tinnitus controls.

	Tinnitus patients (*n* = 58)	Non-tinnitus controls (*n* = 39)	*p* value
Age, years	49.8110.57	47.4611.69	0.307
Gender, male:female	21:37	16:23	0.632
Education levels, years	12.473.09	13.033.04	0.381
Tinnitus duration, months	37.0734.40	−	–
THQ score	52.5013.76	−	–
Hearing thresholds (left)	16.033.02	16.992.48	0.105
Hearing thresholds (right)	16.623.16	16.672.39	0.942
Hearing thresholds (mean)	16.102.64	16.831.51	0.123
**Diabetes characteristics**			
HbA_1c_, % (mmol/mol)	6.531.68	6.151.24	0.228
Fasting glucose, mmol/L	6.482.29	5.791.56	0.103
Triglyceride, mmol/L	1.480.71	1.540.57	0.648
Total cholesterol, mmol/L	5.641.05	5.290.90	0.092
LDL-C, mmol/L	3.440.70	3.240.74	0.201
HDL-C, mmol/L	1.400.32	1.440.34	0.511
**Cognitive performance**			
MMSE	29.071.00	28.771.20	0.187
AVLT	34.027.26	34.106.72	0.954
CFT	34.221.82	34.641.67	0.255
CFT-delayed recall	17.382.49	16.542.98	0.136
TMT-A	64.7220.49	47.7212.84	<0.001*
TMT-B	155.0265.60	103.7236.09	<0.001*
CDT	3.500.54	3.380.59	0.322

Data are presented as mean ± standard deviation. *p < 0.001. THQ, Tinnitus Handicap Questionnaire; LDL-C, low-density lipoprotein cholesterol; HDL-C, high-density lipoprotein cholesterol; MMSE, Mini Mental State Examination; AVLT, Auditory Verbal Learning Test; CFT, Complex Figure Test; TMT-A, Trail Making Test–Part A; TMT-B, Trail Making Test–Part B; CDT, Clock Drawing Test.

In the current study, none of structural changes in specific brain regions were observed between patients with chronic tinnitus and non-tinnitus controls (*p* > 0.05). Besides, the GM, WM, and BP volumes of each group were not significantly different (Table 2). We also did not observe any differences of the brain volumes between tinnitus patients with and without diabetes (*p* > 0.05).

**TABLE 2:** Comparisons of the brain volumes between tinnitus patients and non-tinnitus controls.

	Tinnitus patients (*n* = 58)	Non-tinnitus controls (*n* = 39)	*p* value
Gray matter volume (% of TIV)	31.8 ± 2.0	32.6 ± 2.0	0.071
White matter volume (% of TIV)	29.4 ± 1.5	29.7 ± 1.7	0.448
Brain parenchyma volume (% of TIV)	61.2 ± 2.9	62.2 ± 3.2	0.109

*Data are expressed as mean ± SD. TIV, total intracranial volume.*

**FIGURE 2:**
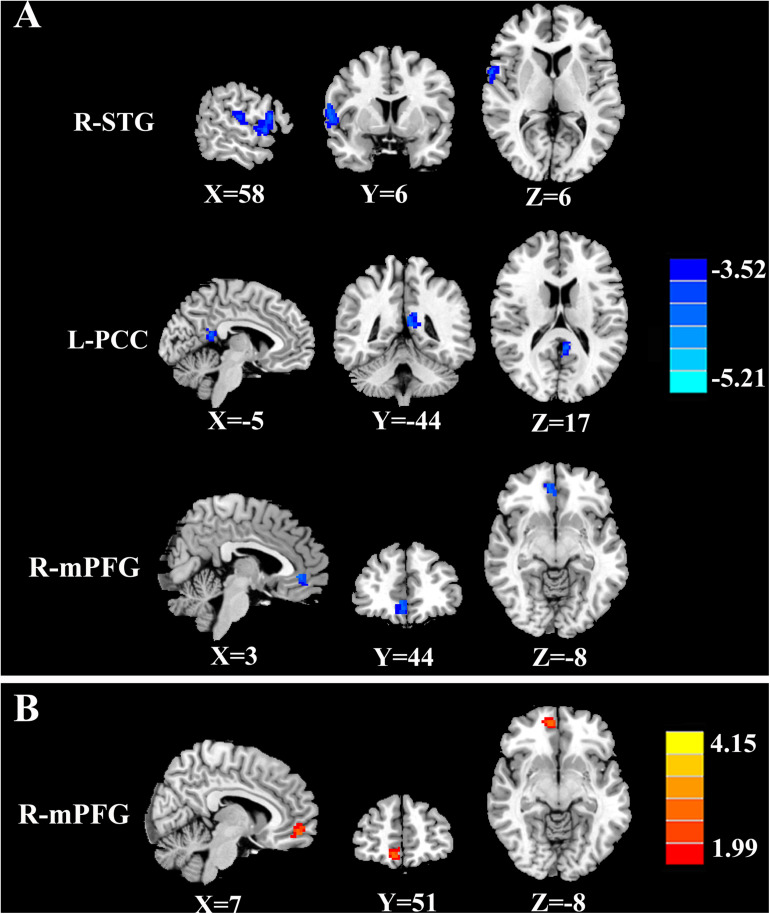
The CBF differences between tinnitus patients and non-tinnitus controls. **(A)** The tinnitus patients exhibited decreased CBF in the right superior temporal gyrus (STG), left posterior cingulate cortex (PCC), and right medial prefrontal gyrus (mPFG) (*p* < 0.01, FWE corrected). **(B)** The interaction effect of diabetes and tinnitus in the right mPFG (*p* < 0.01, FWE corrected).

**FIGURE 3:**
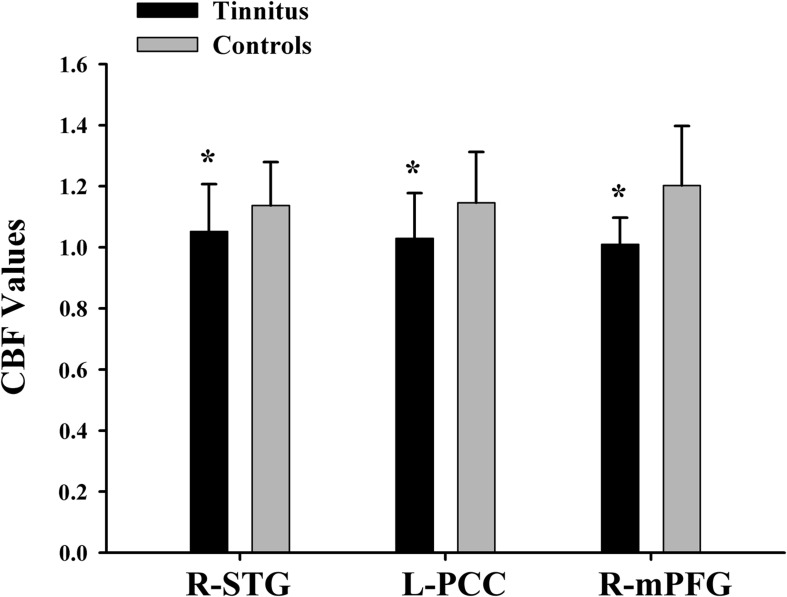
The CBF values of chronic tinnitus patients and non-tinnitus controls in the right STG, left PCC, and right mPFG (*p* < 0.01). *Means significant difference between two groups.

The tinnitus patients displayed disrupted CBF, primarily in the auditory area and DMN, including the decreased CBF regions, namely, right superior temporal gyrus (STG), left posterior cingulate cortex (PCC), and right medial prefrontal gyrus (mPFG) (*p* < 0.01, corrected; Table 3 and Figure 2A). The CBF values in each brain region for different groups are shown in Figure 3. The interaction effect between tinnitus and diabetes was significant in the right mPFG (*p* < 0.01, corrected; Table 4 and Figure 2B). We did not observe other abnormal DMN regions in tinnitus patients with or without diabetes.

**TABLE 3:** Brain regions with significant differences in CBF between tinnitus patients and non-tinnitus controls.

Brain regions	BA	Peak MNI coordinates x, y, z (mm)	Peak *T* value	Cluster size (voxels)
R superior temporal gyrus	22	58, 6, 6	−4.2384	189
L posterior cingulate cortex	23	−5, −44, 17	−4.7829	135
R medial prefrontal gyrus	24	3, 44, −8	−4.1908	140

*Thresholds were set at a corrected *p* < 0.01 corrected by FWE criterion. L, left; R, right.*

**TABLE 4:** Regions showing interaction effects of tinnitus and diabetes on CBF.

Brain regions	BA	Peak MNI coordinates x, y, z (mm)	Peak *T* value	Cluster size (voxels)
R medial prefrontal gyrus	24	7, 51, −8	3.9320	95

*Thresholds were set at a corrected *p* < 0.01 corrected by FWE criterion. R, right.*

**FIGURE 4:**
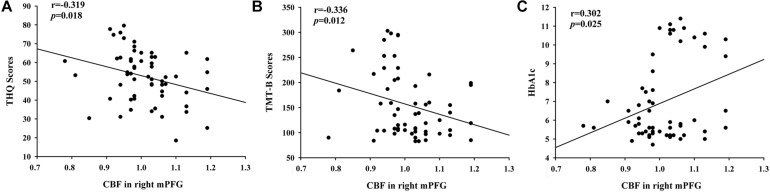
The significant correlations between the CBF changes and the clinical data in tinnitus patients. **(A)** CBF in the right mPFG was negatively associated with THQ scores (*r* = –0.319, *p* = 0.018). **(B)** CBF in the right mPFG was negatively associated with TMT-B scores (*r* = –0.336, *p* = 0.012). **(C)** CBF in the right mPFG was positively correlated with the HbA_1c_ values (*r* = 0.302, *p* = 0.025).

By calculating correlations between CBF in certain areas and THQ scores as well as cognitive scores (Figure 4), we observed that the relative CBF in the right mPFG was correlated THQ scores (*r* = −0.319, *p* = 0.018) and TMT-B scores (*r* = −0.336, *p* = 0.012). Moreover, HbA_1c_ was associated with CBF in the right mPFG in tinnitus patients (*r* = 0.302, *p* = 0.025).

## Discussion

The current study quantified CBF in tinnitus patients and investigated the influence of diabetes on the brain of tinnitus patients. Compared to non-tinnitus controls, tinnitus patients exhibit reduced CBF, which was directly associated with the severity of tinnitus. Interestingly, we also revealed that diabetes exacerbated CBF reduction in tinnitus patients, and poorly controlled glucose level was associated with decreased brain perfusion.

Structural alterations could contribute to aberrant brain perfusion; however, we did not detect structural alterations between tinnitus patients and non-tinnitus subjects in this study. It could be explained by the relatively younger ages and shorter duration of the tinnitus population. Considering the lack of structural change, our results indicated that CBF changes related to tinnitus can occur prior to structural abnormalities.

In our study, main effects of tinnitus were primarily in auditory and DMN regions. Self-directed attention and poor performance on memory tasks could be regarded as symptomatic of a hyperactive autobiographical memory network, known as the DMN in its “resting-state” form, which is responsible for rumination and introspection. The DMN, consisting of nodes in the PCC/anterior cingulate cortex, medial prefrontal cortex, middle temporal cortex, precuneus, and IPL, is active at rest and suspended when entering a task-based state involving attention or goal-directed behavior. Chen et al. investigate the disrupted intrinsic functional connectivity within the DMN regions in tinnitus patients (Chen et al., 2018a), which was correlated with cognitive performance (Chen et al., 2018b). Previous research also implicated a negative influence of tinnitus on verbal fluency, which could be related to a deficit in executive cognitive control (Cardon et al., 2019). Similarly, a previous systematic review indicates the presence of tinnitus negatively impacts the cognitive functioning, particularly attentional functioning (Trevis et al., 2018). Consistent with these, TMT-B test, as a reflection of execution and attention, also showed significant differences between tinnitus patients and controls and is correlated with the diminished CBF in mPFG in the current study. TMT-B score is a neurocognitive test that reflects the function of the prefrontal cortex, and it has been commonly used to define cognitive impairments, especially the executive dysfunction (Ardila et al., 2000). Medial prefrontal cortex takes charge of the ongoing supervision of information and coordinates the activities of multiple cortical regions (Krueger et al., 2007). It is also involved in auditory attention and exerts early inhibitory modulation of input to auditory cortex (Knight et al., 1989). Task MRI study has postulated that the impairment of executive function caused by prefrontal cortex changes could be an important factor in the generation and persistence of tinnitus (Araneda et al., 2017). Previous studies have also suggested the mPFG as an important role in chronic tinnitus (Rauschecker et al., 2015; Araneda et al., 2017; Chen et al., 2018a,b). The relationships between CBF and THQ as well as CBF and TMT-B scores probably support the hypothesis that an abnormal perfusion pattern within DMN and auditory region may play a vital role in tinnitus-related cognitive impairment.

Furthermore, the mPFG works as a vital hub of the DMN and is also considered as a region vulnerable to T2DM. A neuroimaging study focusing on the brain glucose metabolic changes assessed by ^18^F–fluorodeoxyglucose PET reported that the terrible executive function and memory impairment in T2DM correlated with reduced glucose metabolism in the medial prefrontal cortex regions (García-Casares et al., 2014). In another fMRI study exploring the diabetic DMN regions, T2DM patients showed increased connectivity in the medial prefrontal cortex (Cui et al., 2015). In addition, animal experiment also suggested that axon initial segments were significantly shortened in medial prefrontal cortex of db/db mice, a diabetic animal model, compared with controls (Yermakov et al., 2019). In the current study, higher blood glucose levels were correlated with lower CBF values in tinnitus patients, indicating that hyperglycemic state has a negative impact on the brain perfusion in tinnitus patients, and tinnitus patients may benefit from controlled glucose levels to maintain better cognitive functions.

Several limitations of this study should be summarized. First, while we presented some preliminary evidences supporting a possible relationship between the brain perfusion and the cognitive performance, as well as tinnitus distress, it is still inconclusive at this point, and more longitudinal study with bigger sample size of the relationships is required. Second, because of the limited sample size, we did not conduct subgroup analysis. However, the comparison between the left-sided, right-sided, and bilateral tinnitus could be meaningful for the mechanism of the lateralization in tinnitus. Finally, we cannot completely prevent subjects from hearing some sound during the MRI scan, although this study has attempted to minimize the amount of scanner noise with earplugs. It should still be taken into account while attempting to draw conclusions in studies involving auditory stimulation in general.

In conclusion, the current study reveals that patients with tinnitus exhibit disrupted CBF in DMN and auditory regions and that reduced CBF in tinnitus patients associated with the tinnitus distress and cognitive behavior. T2DM exacerbates CBF decline in tinnitus patients, and the better controlled glucose was positively associated with CBF values in tinnitus patients. Thus, tinnitus patients would likely benefit from glucose control in order to maintain brain perfusion and prevent the progression of tinnitus.

## Data Availability Statement

The raw data supporting the conclusions of this article will be made available by the authors, without undue reservation.

## Ethics Statement

The studies involving human participants were reviewed and approved by the Research Ethics Committee of the Nanjing Medical University. The patients/participants provided their written informed consent to participate in this study. Written informed consent was obtained from the individual(s) for the publication of any potentially identifiable images or data included in this article.

## Author Contributions

WX and JC designed the study and supervised the conduct of the study. YL and J-JX contributed to the data collection. HC and XY provided methodological advice, performed the data analysis, and results interpretation. WX, JM, and YW drafted the manuscript, which all authors reviewed and approved for publication.

## Conflict of Interest

The authors declare that the research was conducted in the absence of any commercial or financial relationships that could be construed as a potential conflict of interest.

## Footnotes

1 http://dbm.neuro.uni-jena.de/vbm

2 http://www.fil.ion.ucl.ac.uk/spm/

## References

[B1] Araneda R. Renier L. Dricot L. Decat M. Ebner-Karestinos D. Deggouj N. (2017). A key role of the prefrontal cortex in the maintenance of chronic tinnitus: an fMRI study using a Stroop task. *Neuroimage Clin.* 17 325–334. 10.1016/j.nicl.2017.10.029 29159044 PMC5675730

[B2] Ardila A. Pineda D. Rosselli M. (2000). Correlation between intelligence test scores and executive function measures. *Arch. Clin. Neuropsychol.* 15 31–36. 10.1016/s0887-6177(98)00159-0 14590565

[B3] Ashburner J. Friston K. J. (2005). Unified segmentation. *NeuroImage* 26 839–851. 10.1016/j.neuroimage.2005.02.018 15955494

[B4] Aslan S. Lu H. (2010). On the sensitivity of ASL MRI in detecting regional differences in cerebral blood flow. *Magn. Resonan. Imag.* 28 928–935. 10.1016/j.mri.2010.03.037 20423754 PMC2912434

[B5] Bangen K. J. Werhane M. L. Weigand A. J. Edmonds E. C. Delano-Wood L. Thomas K. R. (2018). Reduced regional cerebral blood flow relates to poorer cognition in older adults with type 2 diabetes. *Front. Aging Neurosci.* 10:270. 10.3389/fnagi.2018.00270 30250430 PMC6139361

[B6] Binnewijzend M. A. Kuijer J. P. Benedictus M. R. Van Der Flier W. M. Wink A. M. Wattjes M. P. (2013). Cerebral blood flow measured with 3D pseudocontinuous arterial spin-labeling MR imaging in Alzheimer disease and mild cognitive impairment: a marker for disease severity. *Radiology* 267 221–230. 10.1148/radiol.12120928 23238159

[B7] Cardon E. Jacquemin L. Mertens G. Van De Heyning P. Vanderveken O. M. Topsakal V. (2019). Cognitive performance in chronic tinnitus patients: a cross-sectional study using the RBANS-H. *Otol. Neurotol.* 40 e876–e882. 31498298 10.1097/MAO.0000000000002403

[B8] Chen Y. C. Chen H. Bo F. Xu J. J. Deng Y. Lv H. (2018a). Tinnitus distress is associated with enhanced resting-state functional connectivity within the default mode network. *Neuropsychiatr. Dis. Treat.* 14 1919–1927. 10.2147/ndt.s164619 30122924 PMC6078076

[B9] Chen Y. C. Zhang H. Kong Y. Lv H. Cai Y. Chen H. (2018b). Alterations of the default mode network and cognitive impairment in patients with unilateral chronic tinnitus. *Quant. Imag. Med. Surg.* 8 1020–1029. 10.21037/qims.2018.11.04 30598879 PMC6288058

[B10] Cui Y. Jiao Y. Chen H. J. Ding J. Luo B. Peng C. Y. (2015). Aberrant functional connectivity of default-mode network in type 2 diabetes patients. *Eur. Radiol.* 25 3238–3246. 10.1007/s00330-015-3746-8 25903712 PMC4595523

[B11] Das S. K. Wineland A. Kallogjeri D. Piccirillo J. F. (2012). Cognitive speed as an objective measure of tinnitus. *Laryngoscope* 122 2533–2538. 10.1002/lary.23555 23108884 PMC3500665

[B12] Detre J. A. Leigh J. S. Williams D. S. Koretsky A. P. (1992). Perfusion imaging. *Magn. Resonan. Med.* 23 37–45. 10.1002/mrm.1910230106 1734182

[B13] Dolui S. Li Z. Nasrallah I. M. Detre J. A. Wolk D. A. (2020). Arterial spin labeling versus 18F-FDG-PET to identify mild cognitive impairment. *NeuroImage Clin.* 25 102146. 10.1016/j.nicl.2019.102146 31931403 PMC6957781

[B14] Farhadi M. Mahmoudian S. Saddadi F. Karimian A. R. Mirzaee M. Ahmadizadeh M. (2010). Functional brain abnormalities localized in 55 chronic tinnitus patients: fusion of SPECT coincidence imaging and MRI. *J. Cereb. Blood Flow Metab.* 30 864–870. 10.1038/jcbfm.2009.254 20068582 PMC2949154

[B15] García-Casares N. Jorge R. E. García-Arnés J. A. Acion L. Berthier M. L. Gonzalez-Alegre P. (2014). Cognitive dysfunctions in middle-aged type 2 diabetic patients and neuroimaging correlations: a cross-sectional study. *J. Alzheimers Dis.* 42 1337–1346. 10.3233/jad-140702 25024335

[B16] Heeren A. Maurage P. Perrot H. De Volder A. Renier L. Araneda R. (2014). Tinnitus specifically alters the top-down executive control sub-component of attention: evidence from the attention network task. *Behav. Brain Res.* 269 147–154. 10.1016/j.bbr.2014.04.043 24793493

[B17] Hu B. Yan L.-F. Sun Q. Yu Y. Zhang J. Dai Y.-J. (2019). Disturbed neurovascular coupling in type 2 diabetes mellitus patients: evidence from a comprehensive fMRI analysis. *NeuroImage Clin.* 22:101802. 10.1016/j.nicl.2019.101802 30991623 PMC6447740

[B18] Husain F. T. Akrofi K. Carpenter-Thompson J. R. Schmidt S. A. (2015). Alterations to the attention system in adults with tinnitus are modality specific. *Brain Res.* 1620 81–97. 10.1016/j.brainres.2015.05.010 25998540

[B19] Khalfa S. Dubal S. Veuillet E. Perez-Diaz F. Jouvent R. Collet L. (2002). Psychometric normalization of a hyperacusis questionnaire. *Orl* 64 436–442. 10.1159/000067570 12499770

[B20] Knight R. T. Scabini D. Woods D. L. (1989). Prefrontal cortex gating of auditory transmission in humans. *Brain Res.* 504 338–342. 10.1016/0006-8993(89)91381-4 2598034

[B21] Krueger F. Moll J. Zahn R. Heinecke A. Grafman J. (2007). Event frequency modulates the processing of daily life activities in human medial prefrontal cortex. *Cereb. Cortex* 17 2346–2353. 10.1093/cercor/bhl143 17190970

[B22] Kuk F. K. Tyler R. S. Russell D. Jordan H. (1990). The psychometric properties of a tinnitus handicap questionnaire. *Ear Hear.* 11 434–445. 10.1097/00003446-199012000-00005 2073977

[B23] Langguth B. Kreuzer P. M. Kleinjung T. De Ridder D. (2013). Tinnitus: causes and clinical management. *Lancet Neurol.* 12 920–930. 10.1016/s1474-4422(13)70160-1 23948178

[B24] Laureano M. R. Onishi E. T. Bressan R. A. Castiglioni M. L. V. Batista I. R. Reis M. A. (2014). Memory networks in tinnitus: a functional brain image study. *PLoS One* 9:e87839. 10.1371/journal.pone.0087839 24516567 PMC3916334

[B25] Lopez-Escamez J. A. Carey J. Chung W.-H. Goebel J. A. Magnusson M. Mandalà M. (2015). Diagnostic criteria for Menière’s disease. *J. Vestib. Res.* 25 1–7. 25882471 10.3233/VES-150549

[B26] Nemati S. Hassanzadeh R. Mehrdad M. Sajedi Kia S. (2018). Hearing status in patients with Type 2 diabetes mellitus according to blood-sugar control: a comparative study. *Iran J. Otorhinolaryngol.* 30 209–218. 30083527 PMC6064765

[B27] Nemholt S. S. Schmidt J. H. Wedderkopp N. Baguley D. M. (2015). Prevalence of tinnitus and/or hyperacusis in children and adolescents: study protocol for a systematic review. *BMJ Open* 5:e006649. 10.1136/bmjopen-2014-006649 25564147 PMC4289724

[B28] Ogoh S. (2017). Relationship between cognitive function and regulation of cerebral blood flow. *J. Physiol. Sci.* 67 345–351. 10.1007/s12576-017-0525-0 28155036 PMC10717011

[B29] Pal K. Mukadam N. Petersen I. Cooper C. (2018). Mild cognitive impairment and progression to dementia in people with diabetes, prediabetes and metabolic syndrome: a systematic review and meta-analysis. *Soc. Psychiatry Psychiatr. Epidemiol.* 53 1149–1160. 10.1007/s00127-018-1581-3 30182156 PMC6208946

[B30] Pierce K. J. Kallogjeri D. Piccirillo J. F. Garcia K. S. Nicklaus J. E. Burton H. (2012). Effects of severe bothersome tinnitus on cognitive function measured with standardized tests. *J. Clin. Exp. Neuropsychol.* 34 126–134. 10.1080/13803395.2011.623120 22168528 PMC3313073

[B31] Rauschecker J. P. May E. S. Maudoux A. Ploner M. (2015). Frontostriatal gating of tinnitus and chronic pain. *Trends Cogn. Sci.* 19 567–578. 10.1016/j.tics.2015.08.002 26412095 PMC4587397

[B32] Taneja N. (2017). Tinnitus, hearing impair-ment and diabetes: a mini-review. *Otolaryngol. Open J*. SE, S6–S9.

[B33] Trevis K. J. Mclachlan N. M. Wilson S. J. (2018). A systematic review and meta-analysis of psychological functioning in chronic tinnitus. *Clin. Psychol. Rev.* 60 62–86. 10.1016/j.cpr.2017.12.006 29366511

[B34] Wang Z. (2012). Improving cerebral blood flow quantification for arterial spin labeled perfusion MRI by removing residual motion artifacts and global signal fluctuations. *Magn. Resonan. Imag.* 30 1409–1415. 10.1016/j.mri.2012.05.004 22789842 PMC3482282

[B35] Xia W. Rao H. Spaeth A. M. Huang R. Tian S. Cai R. (2015). Blood pressure is associated with cerebral blood flow alterations in patients with T2DM as revealed by perfusion functional MRI. *Medicine* 94:e2231. 10.1097/md.0000000000002231 26632913 PMC4674216

[B36] Yermakov L. M. Griggs R. B. Drouet D. E. Sugimoto C. Williams M. T. Vorhees C. V. (2019). Impairment of cognitive flexibility in type 2 diabetic db/db mice. *Behav. Brain Res.* 371:26. 10.1016/j.bbr.2019.111978 PMC6579681 31141724

[B37] Zuloaga K. L. Johnson L. A. Roese N. E. Marzulla T. Zhang W. Nie X. (2016). High fat diet-induced diabetes in mice exacerbates cognitive deficit due to chronic hypoperfusion. *J. Cereb. Blood Flow Metab.* 36 1257–1270. 10.1177/0271678x15616400 26661233 PMC4929700

